# Population Genetic Analyses Reveal the African Origin and Strain Variation of *Cryptococcus neoformans* var. *grubii*

**DOI:** 10.1371/journal.ppat.1002495

**Published:** 2012-02-23

**Authors:** Anastasia P. Litvintseva, Thomas G. Mitchell

**Affiliations:** Department of Molecular Genetics and Microbiology, Duke University Medical Center, Durham, North Carolina, United States of America; Duke University Medical Center, United States of America

*Cryptococcus neoformans* is a ubiquitous, saprobic yeast and the cause of life-threatening infections. Humans acquire the infection by inhaling airborne cells from the environment. In the lungs, these cells become encapsulated yeasts and proliferate. In people with healthy immune responses, the infection may resolve or remain latent and subsequently cause disease. However, in immunocompromised people, such as HIV/AIDS patients, and less often in healthy hosts, the yeasts can disseminate to almost any part of the body; however, they are neurotropic, and meningoencephalitis is the most frequent and deadliest clinical manifestation [1]–[3]. An estimated 1 million new infections are acquired each year, and the majority of these cases occur in sub-Saharan Africa, which has the highest prevalence of patients with HIV/AIDS [4]. In this region, *C. neoformans* is the most common cause of meningitis, and mortality hovers around 50%. Others who succumb to cryptococcosis are apparently immunocompetent and exhibit no evidence of underlying disease. For example, 71% of cryptococcal infections in China occur in people without pre-existing conditions [5].

There are two varieties, *C. neoformans* var. *grubii* (*Cng*) and *C. neoformans* var. *neoformans* (*Cnn*), which are distinguishable by molecular markers or their capsular serotypes, A or D, respectively. Diploid AD hybrids also occur in the environment and patients [6]–[8]. In addition, a sibling species, *Cryptococcus gattii*, causes similar infections. However, isolates of both serotype D and AD hybrids, as well as *C. gattii*, are much less common. At least 90% of human cryptococcal disease and fatalities are caused by *Cng* (serotype A) [9]–[11].

## Non-African Global Isolates of *Cng* Are Highly Clonal

Strains of *Cng* have been isolated from all continents except Antarctica. Molecular epidemiological studies identified significant clonality among global strains, as strains with identical molecular genotypes have been isolated from different geographic areas, continents apart [5], [12], [13]. The use of reproducible and robust multilocus sequence typing (MLST) has determined that the overwhelming majority of non-African strains of *Cng* are represented by only a few genotypes [13]–[15].

Isolates from southeastern Asia are remarkably homogeneous. For example, all the clinical isolates from a cohort of 120 Chinese patients were infected with the same cosmopolitan MLST genotype, M5 [5]. Similarly, all seven clinical isolates from Japan [13] and 70 of 75 from South Korea possessed the M5 genotype (designated “VNIc” in the original paper) [16]. In Thailand, 183 clinical and environmental isolates were analyzed, and 96% of the isolates were represented by only three MLST genotypes, one of which was M5 (designated “ST46” in the original paper) [15].

In the United States, an analysis of over 800 isolates yielded only ten distinct genotypes, and M5 was the most prevalent among both clinical and environmental samples (designated “A5” in the original paper) [10]. In comparison, five genotypes were found in Europe, and nine genotypes among isolates from central and eastern Africa, but only two different MLST genotypes were detected from South American and Australian isolates, although these were small samples [13]. More recently, we found that M5 was the most prevalent genotype among isolates from patients with recurrent cryptococcosis in South Africa (unpublished data), even though, as described below, this region has the highest overall genetic diversity.

## Southern African Isolates of *Cng* Are Highly Diverse

Unlike the rest of the world, southern Africa harbors a geographically restricted, genetically diverse population of *Cng*. In 2003, isolates from 200 HIV-seropositive patients in Botswana were shown to possess novel genotypes that differed from isolates found anywhere else. Analyses of mating indicated that 12% of these strains possess the *MAT* **a** mating type, which is exceedingly rare among non-African strains, and population genetic analysis demonstrated evidence for recombination in this population [17]. Subsequent environmental sampling confirmed that two genetically isolated subpopulations are localized in southern Africa: (i) a genetically diverse, endemic population that is restricted to southern African and associated with indigenous African trees, especially the mopane tree (*Colophospermum mopane*), and (ii) a cosmopolitan population of strains with molecular types that are found worldwide and frequently associated with the excreta of feral pigeons (*Columba livia*) [14].

Population genetic analyses of the environmental strains revealed limited genetic interaction between the endemic (arboreal) and cosmopolitan (coprophilic) populations. The arboreal population was characterized by linkage equilibrium among loci and high genetic diversity, which can be explained by recombination, ancestral origin, or both. However, when putative recombinant haplotypes were removed from the analysis, significantly high indices of genetic diversity were still detected in the African population [14].

## Phylogenetic Analyses Indicate That African Strains of *Cng* Possess Ancestral Haplotypes

**Figure 1:**
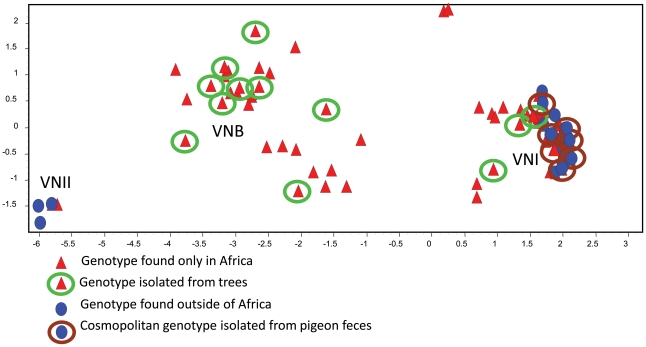
The genetic relationships among MLST genotypes are visualized by PCA. Each symbol represents a genotype with a unique eight-digit allelic profile. Red triangles represent genotypes of strains that are endemic to Africa, and blue circles represent genotypes of global strains. Genotypes associated with African trees are enclosed in green circles, and genotypes associated with pigeon excreta are enclosed in brown circles. Genotypes without circles represent clinical strains that to date have not been isolated from the environment. (From reference [14] and used with permission of the publisher.)

Phylogenetic analysis and principal component analysis (PCA) of the MLST profiles indicate that *Cng* is comprised of three isolated subpopulations, VNI, VNII, and VNB (Figure 1). The global population is markedly clonal and consists of VNI and VNII strains with few unique genotypes. The arboreal, southern African population is geographically confined and comprised of almost all the known VNB strains and a large number of VNI strains that are genetically diverse. Thus, multiple features of the African strains—greatest genetic diversity, prevalence of both mating types, and association with an indigenous reservoir—suggest they represent the ancestral origin of *Cng*.

**Figure 2:**
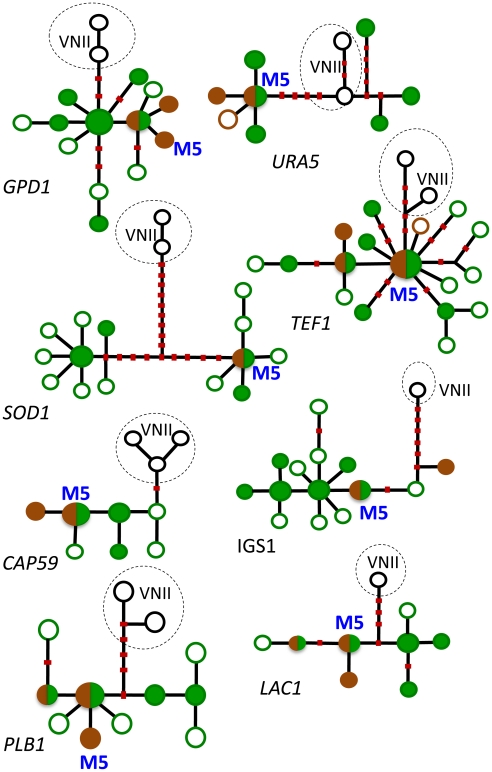
Haplotype networks of the eight MLST loci. Haplotypes of strains of *Cng* that have never been found outside Africa are shown in green: filled green circles designate haplotypes of strains that were obtained from trees (most were also found in patients), and empty green circles signify haplotypes that were obtained only from patients. Cosmopolitan haplotypes are shown in brown: filled brown circles designate haplotypes of strains from pigeon excreta (most were also found in patients), and empty brown circles signify haplotypes that were obtained only from patients. Circles that are half green and half brown indicate haplotypes of strains found in trees and pigeon excreta. Haplotypes from the global VNII subpopulation of *Cng* are included as an outgroup; they are shown in black and lightly encircled. Red dots on the lines connecting the haplotypes represent the most parsimonious number of mutational steps required to generate the allelic polymorphisms. Recombinant haplotypes are excluded. The most common haplotype in Asia and elsewhere, M5, is shown in blue. (From reference [14] and used with permission of the publisher.)

This conclusion was supported by phylogenetic analyses of individual loci using methods of maximum likelihood and parsimony. To illustrate, haplotype networks were analyzed by statistical parsimony to infer any phylogenetic relationships among the haplotypes. The internal nodes of these networks represent ancestral haplotypes from which the distal, derived haplotypes evolved. Numerous haplotypes from the endemic African population occupied both internal (ancestral) and distal (derived) positions on the networks (Figure 2, green circles). Conversely, haplotypes that are unique to the global population were scarce and almost always in distal positions, which suggests they originated more recently. All of these genotypes were obtained from pigeon habitats (Figure 2, brown circles) [14], [15]. Furthermore, maximum likelihood analyses of three loci (*TEF1*, *CAP59*, and *PLB1*) indicated that the ancestral haplotypes of both the VNI and VNB populations are confined to southern Africa and associated with endemic African trees [14].

## Evidence That Pigeons Facilitated the Global Dispersion of Southern African Strains

Environmental sampling has demonstrated that African strains of VNI and VNB are associated with native African trees, whereas cosmopolitan strains of VNI are isolated from pigeon droppings. VNI strains with identical MLST genotypes have been isolated from pigeon habitats in North and South America, Europe, Asia, and Africa [10], [13], [14]. Coalescent simulations estimated that the cosmopolitan and African populations diverged approximately 5,000 years ago, which is around the postulated time period when *C. livia* (rock doves or pigeons) were domesticated [15]. Although the exact origin of *C. livia* is unknown, historical records indicate that pigeons were probably native to the north African Mediterranean region and were spread globally over the last 400 years of European expansion [18], [19]. Thus, multiple features of the cosmopolitan population of VNI—exceptionally low genetic diversity, dominance of a single mating type, and global distribution in association with pigeon excreta—support the parsimonious conclusion that pigeons facilitated the global exportation and dispersion of African strains.

## Phenotypic Diversity among Isolates of *Cng*

Cryptococcal virulence is complex and polygenic, involving dozens of genes and signal transduction pathways [20]. Several studies have documented variation among strains of *Cng* in the expression of virulence phenotypes, such as the size, composition, and biological activity of the capsule, susceptibility to antifungal drugs, resistance to phagocytes, and others [21]–[25]. This marked phenotypic variation among wild-type isolates of *Cng* suggests that some strains are inherently more virulent. As observed in several reports and noted above, patients may survive or succumb to cryptococcosis regardless of their immune status, which suggests that the virulence of an infecting strain may be as important as the host's defenses. Does the genotype of a strain impart any clinical relevance? A recent population genetics investigation used 11 MLST markers to genotype isolates from South African pediatric cases of cryptococcosis [26]. Seventy-one children, nearly all HIV-positive, were infected with 17 different VNI genotypes; most genotypes were equally present in boys and girls, but one genotype was significantly more prevalent in boys [26]. When the relevance of genotype was tested experimentally, clinical and environmental isolates with identical MLST profiles varied dramatically in murine virulence; however, virulence was associated with the clinical or environmental source of a strain rather than its genotype [27]. Clearly, strains with the same genotype exhibit phenotypic variation. Nevertheless, some genotypes are highly prevalent in the environment but rare or absent in patients and vice versa [10], [28]. The most important phenotype is the production of disease in people. With the advent of next-generation sequencing, it is now possible to use MLST genotypes to select strains differing in genetic diversity and clinical prevalence and to conduct comparative genomic analyses to address the question of why some strains are more pathogenic.

## References

[1] Lin X, Heitman J (2006). The biology of the *Cryptococcus neoformans* species Complex.. Annu Rev Microbiol.

[2] Ma H, May RC (2009). Virulence in *Cryptococcus* species.. Adv Appl Microbiol.

[3] Perfect JR, Dismukes WE, Dromer F, Goldman DL, Graybill JR (2010). Clinical practice guidelines for the management of cryptococcal disease: 2010 update by the Infectious Diseases Society of America.. Clin Infect Dis.

[4] Park BJ, Wannemuehler KA, Marston BJ, Govender N, Pappas PG (2009). Estimation of the current global burden of cryptococcal meningitis among persons living with HIV/AIDS.. AIDS.

[5] Chen J, Varma A, Diaz MR, Litvintseva AP, Wollenberg KK (2008). *Cryptococcus neoformans* strains and infection in apparently immunocompetent patients, China.. Emerg Infect Dis.

[6] Lengeler K, Cox G, Heitman J (2001). Serotype AD strains of *Cryptococcus neoformans* are diploid or aneuploid and heterozygous at the mating-type locus.. Infect Immun.

[7] Litvintseva AP, Lin X, Templeton I, Heitman J, Mitchell TG (2007). Many globally isolated AD hybrid strains of *Cryptococcus neoformans* originated in Africa.. PLoS Pathog.

[8] Xu J, Luo G, Vilgalys RJ, Brandt ME, Mitchell TG (2002). Multiple origins of hybrid strains of *Cryptococcus neoformans* with serotype AD.. Microbiology.

[9] Govender N, Mitchell TG, litvintseva AP, Miglia KJ, Heitman J, Kozel TR, Kwon-Chung J, Perfect JR, Casadevall A (2011). Cryptococcosis in Africa.. *Cryptococcus:* from human pathogen to model yeast.

[10] Litvintseva AP, Kestenbaum L, Vilgalys R, Mitchell TG (2005). Comparative analysis of environmental and clinical populations of *Cryptococcus neoformans*.. J Clin Microbiol.

[11] Xu J, Manosuthi W, Banerjee U, Zhu L-P, Chen J, Heitman J, Kozel TR, Kwon-Chung J, Perfect JR, Casadevall A (2011). Cryptococcosis in Asia.. *Cryptococcus* from human pathogen to model yeast.

[12] Bovers M, Hagen F, Kuramae EE, Boekhout T (2008). Six monophyletic lineages identified within *Cryptococcus neoformans* and *Cryptococcus gattii* by multi-locus sequence typing.. Fungal Genet Biol.

[13] Litvintseva AP, Thakur R, Vilgalys R, Mitchell TG (2006). Multilocus sequence typing reveals three genetic subpopulations of *Cryptococcus neoformans* var. *grubii* (serotype A), including a unique population in Botswana.. Genetics.

[14] Litvintseva AP, Carbone I, Rossouw J, Thakur R, Govender NP (2011). Evidence that the human pathogenic fungus *Cryptococcus neoformans* var. *grubii* may have evolved in Africa.. PLoS ONE.

[15] Simwami SP, Khayhan K, Henk DA, Aanensen DM, Boekhout T (2011). Low diversity *Cryptococcus neoformans* variety *grubii* multilocus sequence types from Thailand are consistent with an ancestral African origin.. PLoS Pathog.

[16] Choi YH, Ngamskulrungroj P, Varma A, Sionov E, Hwang SM (2010). Prevalence of the VNIc genotype of *Cryptococcus neoformans* in non-HIV-associated cryptococcosis in the Republic of Korea.. FEMS Yeast Res.

[17] Litvintseva AP, Marra RE, Nielsen K, Heitman J, Vilgalys R (2003). Evidence of sexual recombination among *Cryptococcus neoformans* serotype A isolates in sub-Saharan Africa.. Eukaryot Cell.

[18] Long JL (1981). Introduced birds of the world: the worldwide history, distribution and influence of birds introduced to new environments.

[19] Grzimek B, Schlager N, Olendorf D, McDade MC (2004). Pigeons and doves.. Grzimek's animal life encyclopedia. Volume 9: Birds II.

[20] Kronstad JW, Attarian R, Cadieux B, Choi J, D'Souza CA (2011). Expanding fungal pathogenesis: *Cryptococcus* breaks out of the opportunistic box.. Nat Rev Microbiol.

[21] Kumar P, Yang M, Haynes BC, Skowyra ML, Doering TL (2011). Emerging themes in cryptococcal capsule synthesis.. Curr Opin Struct Biol.

[22] Liaw SJ, Wu HC, Hsueh PR (2010). Microbiological characteristics of clinical isolates of *Cryptococcus neoformans* in Taiwan: serotypes, mating types, molecular types, virulence factors, and antifungal susceptibility.. Clin Microbiol Infect.

[23] Mitchell TG, Litvintseva AP, Ashbee HR, Bignell EM (2010). Typing species of *Cryptococcus* and epidemiology of cryptococcosis.. Pathogenic yeasts.

[24] Pedroso RS, Lavrador MA, Ferreira JC, Candido RC, Maffei CM (2010). *Cryptococcus neoformans* var. *grubii* - pathogenicity of environmental isolates correlated to virulence factors, susceptibility to fluconazole and molecular profile.. Mem Inst Oswaldo Cruz.

[25] Alanio A, Desnos-Ollivier M, Dromer F (2011). Dynamics of *Cryptococcus neoformans*-macrophage interactions reveal that fungal background influences outcome during cryptococcal meningoencephalitis in humans.. mBio.

[26] Miglia KJ, Govender NP, Rossouw J, Meiring S, Mitchell TG (2011). Analyses of pediatric isolates of *Cryptococcus neoformans* from South Africa.. J Clin Microbiol.

[27] Litvintseva AP, Mitchell TG (2009). Most environmental isolates of *Cryptococcus neoformans* var. *grubii* (serotype A) are not lethal for mice.. Infect Immun.

[28] Illnait-Zaragozi MT, Martinez-Machin GF, Fernandez-Andreu CM, Boekhout T, Meis JF (2010). Microsatellite typing of clinical and environmental *Cryptococcus neoformans* var. *grubii* isolates from Cuba shows multiple genetic lineages.. PLoS ONE.

